# Propionic Acid, Induced in Gut by an Inulin Diet, Suppresses Inflammation and Ameliorates Liver Ischemia and Reperfusion Injury in Mice

**DOI:** 10.3389/fimmu.2022.862503

**Published:** 2022-04-22

**Authors:** Junya Kawasoe, Yoichiro Uchida, Hiroshi Kawamoto, Tomoyuki Miyauchi, Takeshi Watanabe, Kenichi Saga, Kosuke Tanaka, Shugo Ueda, Hiroaki Terajima, Kojiro Taura, Etsuro Hatano

**Affiliations:** ^1^ Department of Surgery, Graduate School of Medicine, Kyoto University, Kyoto, Japan; ^2^ Department of Gastroenterological Surgery and Oncology, Kitano Hospital Medical Research Institute, Osaka, Japan; ^3^ Division of Immunology, Institute for Frontier Life and Medical Sciences, Kyoto University, Kyoto, Japan

**Keywords:** liver ischemia-reperfusion injury, inulin diet, short-chain fatty acid, gut microbiota, propionic acid

## Abstract

Liver ischemia and reperfusion injury (IRI) is one of the obstacles in liver surgery such as liver resection and transplantation. In this study, we investigated the preventive effect on mouse liver IRI by feeding mice with inulin, which is a heterogeneous blend of indigestible fructose polymer. Mice were fed either a control ordinary diet (CD) or an inulin diet (ID) containing 5% inulin in the CD, for 14 days before the ischemia and reperfusion (IR) maneuver. IR induced-liver damages were significantly ameliorated in the ID group, compared with those in the CD group. Feeding mice with an ID, but not a CD, elevated levels of *Bacteroidetes* among gut microbiota, and especially increased *Bacteroides acidifaciens* in mouse feces, which resulted in significant elevation of short-chain fatty acids (SCFAs) in the portal vein of mice. Among SCFAs, propionic acid (PA) was most significantly increased. The microbial gene functions related to PA biosynthesis were much higher in the fecal microbiome of the ID group compared to the CD. However, the action of PA on liver IRI has not been yet clarified. Direct intraperitoneal administration of PA alone prior to the ischemia strongly suppressed liver cell damages as well as inflammatory responses caused by liver IR. Furthermore, PA suppressed the secretion of inflammatory cytokines from peritoneal macrophages stimulated *in vitro* through TLR-4 with high-mobility group box 1 protein (HMGB-1), known to be released from apoptotic liver cells during the IR insult. The present study shows that PA may play a key role in the inulin-induced amelioration of mouse liver IRI.

## Introduction

Liver ischemia and reperfusion injury (IRI) is an inevitable event in the field of liver surgery including liver transplantation, and it has been a major cause of postoperative liver dysfunction and failure (1, 2). IRI is caused by various mechanisms, including the release of inflammatory cytokines and chemokines that results in the activation of neutrophils and macrophages. As to liver IRI, damage-associated molecular patterns (DAMPs) such as high-mobility group box 1 (HMGB-1) proteins, free fatty acids, and heat shock proteins are released from damaged liver sinusoidal endothelial cells and hepatocytes (3). These molecules increase and amplify ischemia and reperfusion (IR)-induced liver injury. Reactive oxygen species (ROS) generated by activated Kupffer cells are also a major mediator in liver IRI (4). So far, we have reported various approaches to overcome liver IRI (5–7). It has been shown that HMGB-1 released from an IR-injured liver acts through the Toll-like receptor 4 (TLR-4) on hepatocytes and macrophages, which results in the apoptotic cell death of hepatocytes and induction of severe inflammatory responses by macrophages (8). Blocking the interaction between HMGB-1 and TLR-4 could prevent liver IRI in a TLR-4 dependent manner (5, 6). Recently, the Enhanced Recovery After Surgery (ERAS) protocol is advocated in the field of liver surgery. ERAS is a multifaceted pathway, including a preoperative nutritional approach, and has been developed to overcome the deleterious effect of perioperative stress after surgery (9). We recently reported a finding that pretreatment of mice by the short-term (12 h) restriction of feeding remarkably ameliorates IR-induced liver damage and inflammation (7).

Inulin is a water-soluble fermentable dietary fiber, belonging to a group of nondigestible carbohydrates called fructans (10). Inulin cannot be absorbed by the human intestinal tract, and is decomposed by gut microbiota into degradation products, such as short-chain fatty acids (SCFAs) (11, 12). SCFAs interact with various receptors, such as G protein-coupled receptors (GPRs), GPR41, GPR43, and GPR109A, expressed on the gut epithelium and immune cells (13). The interaction between SCFAs and GPRs is related to the activity of immune cells and the induction of regulatory T cells. Butyrate is one of the SCFAs and has been reported to induce the differentiation of regulatory T cells and to ameliorate the development of colitis (14). The effects of SCFAs, such as butyric acid, propionic acid (PA), and valeric acid, have been reported to show a negative correlation with the immunostimulatory M1 macrophages, but a positive correlation with the immunoinhibitory M2 macrophages (15). In a mouse alcoholic liver disease model, feeding mice with inulin ameliorates the inflammation by inducing the suppression of M1 and facilitating M2 macrophages *via* SCFAs (15). Various previous reports suggest the efficacy of SCFAs, especially butyric acid, against IR-induced organ injury (16–22). In the present study, we show, for the first time, that feeding mice with an inulin-rich enteral diet significantly reduced mouse liver IR insult, suggesting the efficacy of preoperative inulin-rich enteral diets against liver IRI as a novel means of ERAS. PA among the SCFAs, was most significantly increased in the portal vein of mice fed with an inulin-rich diet, suggesting that inulin diet efficiently promoted the production of PA by gut microbiota. Although few studies thus far have reported the efficacy of PA on liver IRI, the present study clearly shows that the intraperitoneal administration of PA alone resulted in remarkable amelioration of liver injury through not only the protection from liver cell death caused by IR but also the suppression of proinflammatory responses induced in macrophages activated by HMGB-1. PA might play a key role in the inulin diet-induced suppression of liver IRI.

## Materials and Methods

### Animals

Male C57BL/6 mice (8-10 weeks old, weighing 20-25 g) were purchased from Shimizu Laboratory Supplies (Kyoto, Japan). All animals were maintained in specific pathogen-free conditions and received humane care according to the Guide for Care and Use of Laboratory Animals. All experimental protocols were approved by the Animal Research Committee of The Tazuke Kofukai Medical Research Institute, Kitano Hospital, Osaka, Japan.

### Mouse Diets

The control ordinary diet (CD) was AIN-93G (23). The inulin diet (ID) was the CD supplemented with 5% inulin. We determined the content of inulin based on the previous studies (24, 25). Both diet reagents were provided by EN Otsuka Pharmaceutical Co., Ltd. (Hanamaki, Iwate, Japan). The compositions of both diets are shown in **Table 1**.

**Table 1:** Compositions of the Control ordinary diet and Inulin diet.

	Control ordinary diet (AIN-93G)	Inulin diet
	(377 kcal/100g)	(366 kcal/100g)
	(%)	(%)
Casein	20.0000	20.0000
L-cystine	0.3000	0.3000
**Corn starch**	**39.7486**	**34.7486**
αcorn starch	13.2000	13.2000
Sucrose	10.0000	10.0000
Soybean oil	7.0000	7.0000
Cellulose powder	5.0000	5.0000
Minerals	3.5000	3.5000
Vitamins	1.0000	1.0000
Choline Bitartrate	0.2500	0.2500
t-butylhydroquinone	0.0015	0.0014
**inulin**	**0.0000**	**5.0000**

The bold values mean our intention to emphasize the difference between the two groups.

### Liver IRI Model

We used the established mouse model of partial warm liver IRI (5, 6). Mice were anesthetized under isoflurane and injected with heparin (100 U/kg). An atraumatic clip was used to interrupt the artery and portal venous supply and the bile duct to the left and middle liver lobes. After 60 minutes of ischemia at room temperature, the clamp was removed, and reperfusion was initiated. After 6 hours of reperfusion, mice were sacrificed. Liver samples were immediately fixed overnight in 10% formaldehyde or frozen in liquid nitrogen until the extraction procedure. Sham-operated mice underwent the same procedure, but without vascular occlusion.

### Hepatocyte Function

Serum alanine aminotransferase (sALT) levels, used as a measure of liver injury, were determined by a standard spectrophotometric method with an automated clinical analyzer (JCABM9030, JEOL Ltd., Tokyo, Japan).

### Treatment of Mice With Propionic Acid

Propionic acid (PA) solution was purchased from FUJIFILM Wako Pure Chemical Corporation (Osaka, Japan) and diluted with saline. After sterilization by filter, PA (at 5 mmol/kg, 100μM per a mouse of 20 grams weight, in saline) was administered intraperitoneally 2 hours before the ischemic insult. The dosage of PA was determined based on the previous report (26).

### Histology

Liver paraffin sections (5 µm thick) were stained with hematoxylin and eosin (H&E). The severity of liver IRI (necrosis, sinusoidal congestion, and centrilobular ballooning) was graded on a scale from 0 to 4 by an investigator who was blinded to the experimental conditions using the modified Suzuki’s criteria (27). In the modified Suzuki’s criteria, scores are determined by the presence of congestion (from a value of 0 to a value of 4 depending on the severity), vacuolization (from 0 to 4) and necrosis (from 0 to 4). Ten fields were examined for each sample.

### Enzyme-Linked Immunosorbent Assay (ELISA)

The serum HMGB-1 level was quantified with the HMGB-1 ELISA Kit II (Shino-Test, Tokyo, Japan). The medium from peritoneal macrophage culture was analyzed for tumor necrosis factor (TNF)-α using an ELISA kit as per the manufacturer’s instructions (R&D Systems, Minneapolis, MN, USA).

### Western Blot Assay

Western immunoblotting was performed using standard techniques as described previously (7). Primary antibodies used are listed in **Table S1**.

### Quantitative Reverse-Transcription Polymerase Chain Reaction (qRT-PCR)

Total RNA was extracted from liver tissues with the RNeasy Mini Kit (QIAGEN, Venlo, the Netherlands). Complementary DNA was prepared using a PrimeScript RT Reagent Kit (TAKARA BIO, Kusatsu, Japan). qRT-PCR was performed using the StepOnePlus_TM_ Real-Time PCR System (Life Technologies, Tokyo, Japan). Primers used to amplify specific gene fragments are listed in **Table S2**. Target gene expression was calculated using the ratio of that gene to the housekeeping gene, β-actin.

### Apoptosis Assay

Apoptosis in 5-µm-thick liver paraffin sections was detected by the terminal deoxynucleotidyl transferase-mediated deoxyuridine triphosphate nick-end labeling (TUNEL) method using an *in situ* Apoptosis Detection Kit (Takara Bio, Kyoto, Japan), according to the manufacturer’s instructions.

### Analyses of Gut Microbiota in Mice

Mice were divided into two groups. Mice in each group were fed a CD or an ID for 14 days before feces collection. On day 15, all fecal samples obtained from mice of both groups were collected and stored at -80 °C until analyses of the gut microbial flora. A two-step tailed PCR method was used for the preparation of dsDNA libraries. Library concentrations were measured with a Synergy H1 microplate reader (BioTek) and a QuantiFluor dsDNA System (Promega). The library quality was assessed using a Fragment Analyzer (Advanced Analytical Technologies, Ankeny, IA, USA) with a dsDNA 915 Reagent Kit (Agilent, Santa Clara, CA, USA). Paired-end sequencing (2 × 300 bp) was performed on the Illumina MiSeq platform (Illumina, San Diego, CA, USA) with the MiSeq Reagent Kit v3 (Illumina). A sequence that completely matched the primer used was extracted by using the fast barcode splitter tool. After the trimming of the primer sequence, denoized sequences were analyzed using Qiime2.0 (2019.4). The EzBioCloud 16S database (28) was used to classify the bacterial species. These analysis procedures were performed at Bioengineering Lab. Co., Ltd. (Sagamihara, Kanagawa, Japan).

### Predictive Function Analysis

Phylogenetic Investigation of Communities by Reconstruction of Unobserved States (PICRUSt) (29) provided a number of scripts in fecal microbiome, that could be useful for analyzing both 16S rRNA gene relative abundances and the predicted metabolic data.

### Quantification of SCFAs in the Portal Vein

After mice were fed with a CD or an ID for 14 days, the blood in the portal vein of mice in both groups were sampled with 27-gauge needles. After centrifugation at 2000 × g for 10 minutes at room temperature, serum samples were collected and stored at -80°C until the detection of SCFAs. The SCFA extraction procedure and analysis methods were the same as those described previously by the Kyoto Institute of Nutrition and Pathology (Kyoto, Japan) (30, 31).

### Cell Cultures

Thioglycollate-elicited peritoneal macrophages, which were collected using a method described previously (5), were plated into 24-well cell culture plates, at 2.0 × 10^5^ cells/well at a volume of 0.5 mL/well, and the plates were incubated in a humidified atmosphere of 5% CO_2_ and 95% air. After 3 hours, adherent cells were recovered and washed three times with phosphate-buffered saline to remove non-adherent cells. Resultant peritoneal macrophages were then incubated with various concentrations of PA (n = 3 in each group) for 30 minutes. Then, bovine HMGB-1 (Chondrex, Redmond, WA, USA) (1 µg/mL) was added to the macrophages. Forty-eight hours after treatment, supernatants were collected and stored at -80°C until measurements of TNF-α were performed by ELISA. Plated macrophages were collected by dissolving in cell lysis buffer with protease and phosphorylation inhibitors. Cell lysates were stored at -80°C until western blotting analyses.

### Flow Cytometry

The ratio of Foxp3-positive T cells to CD4-positive cells was assessed by flow cytometry using CytoFLEX (Beckman Coulter, Brea, CA, USA). The FOXP3 Fix/Perm Buffer Set (BioLegend, San Diego, CA, USA) was used for the intracellular staining of Foxp3. Anti-CD4 monoclonal antibody (FITC) and anti-Foxp3 monoclonal antibody (APC) were purchased from eBioscience (San Diego, CA, USA).

### Statistical Analyses

All data are presented as the means ± standard deviations. Differences between experimental groups were analyzed using the one-way analysis of variance of Student’s t-test for unpaired data. All differences were considered statistically significant at a P-value less than 0.05.

## Results

### Two Weeks of Feeding With an ID Suppressed Liver IRI

**Figure 1:**
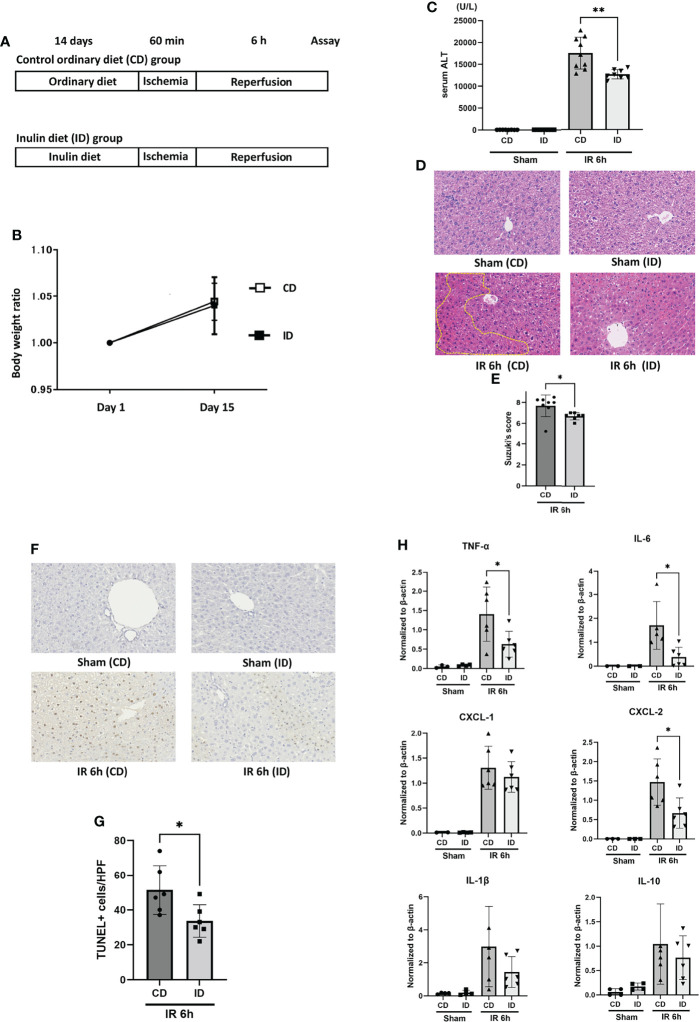
Feeding with an inulin diet ameliorates liver ischemia and reperfusion injury in mice. **(A)** Wild-type (WT) mice were divided into two groups. They were fed a CD or an ID for 14 days prior to IR stimulation. Then, mice in both groups were subjected to liver IRI treatment which means 60 min of liver ischemia, followed by 6 h of reperfusion. After 6 h of reperfusion, the serum and liver tissue of each mouse was collected for assays. Sham-operated mice underwent the same procedure, but without vascular occlusion. **(B)** The body weight of each mouse was monitored on days 1 and 15. **(C)** The sALT level after 6 h of reperfusion (n = 8 mice/group; **P < .01) (Power calculation: 1.000). **(D)** Representative liver histology (H&E staining) after IR insult (magnification ×400). Congestion area was shown in circle by yellow dot. **(E)** Suzuki’s histological grading in each group (n = 8 mice/group; *P < .05). **(F)** Representative TUNEL-assisted detection of hepatic apoptosis in liver tissues after IR (magnification ×400). **(G)** Quantification of hepatic apoptosis by counting TUNEL-positive cells. (n = 8 mice/group; *P < .05). (Power calculation: 0.981) **(H)** Quantitative RT-PCR detection of inflammatory cytokines (TNF-α, IL-6, CXCL-1, CXCL-2, IL-1β, and IL-10) at 6 h of reperfusion. Data were normalized to β-actin gene expression (n = 6 mice/group; *P < .05) (Power calculation: TNF-α; 0.968, IL-6; 0.984, CXCL-2; 0.990).

The control ordinary diet (CD) was AIN-93G (23). The inulin diet (ID) was the CD supplemented with 5% inulin (**Table 1**). We determined the content of inulin based on the previous studies (24, 25). It has been reported that feeding with AIN-93G plus 5% inulin could reduce the presence of Clostridium XI in mouse intestinal microbiota (25). The two groups of mice were fed either a CD or an ID, in addition to water ad libitum, for 14 days prior to IR stimulation. Then, mice in both groups received the liver IR treatment (**Figure 1A**). The body weights of the mice in both groups before IR were measured on days 1 and 15 after feeding them with the CD or ID. No significant difference in body weight was observed in the two groups (**Figure 1B**). The sALT titers of mice in the ID group after liver IR insult were decreased significantly compared with those in the CD group (**Figure 1C**). As **Figure 1D** shows, liver histology displayed prominent hepatocellular necrosis, in addition to congestion and ballooning, among the mice in the CD group after IR stimulation. By contrast, the livers from the ID group subjected to IR stimulation revealed significantly less pathology, as shown in **Figure 1D**. Suzuki’s score was also significantly lower in the ID group (**Figure 1E**). As IR stimulation has been reported to promote the production of ROS and hepatocyte apoptosis (4, 32, 33), we examined liver tissues from both groups using the TUNEL assay to assess apoptosis. The numbers of TUNEL-positive cells induced by IR were diminished significantly in the ID group compared with the CD group (**Figures 1F, G**). It has been reported that inflammatory cytokines and chemokines play a key role in liver IRI (34, 35). Feeding mice with an ID resulted in a significant decrease in the expression of proinflammatory cytokines, such as TNF-α and IL-6. As to chemokines, CXCL-2 expression was strongly suppressed in the ID group (**Figure 1H**), suggesting that the infiltration of neutrophils into the liver may also be reduced. However, no significant increase was observed in the expression of immunosuppressive cytokines, such as IL-10, in liver tissues from the ID group (**Figure 1H**). The ratio of Foxp3-positive T cells in the mesenteric lymph nodes showed no significant difference between both groups (**Figure S1**). These results indicated that the inulin-enriched diet could exhibit the amelioration of liver injury induced by IR through the suppression of proinflammatory cytokines and the prevention of apoptosis. However, either immunosuppressive cytokines, such as IL-10 or Foxp3-positive regulatory T cells appeared not to play a crucial role in the suppression of inflammation as well as in the improvement of liver cell survival during IRI.

### Effects of ID on Distribution of Gut Microbiota and SCFA Concentration in the Portal Vein

**Figure 2:**
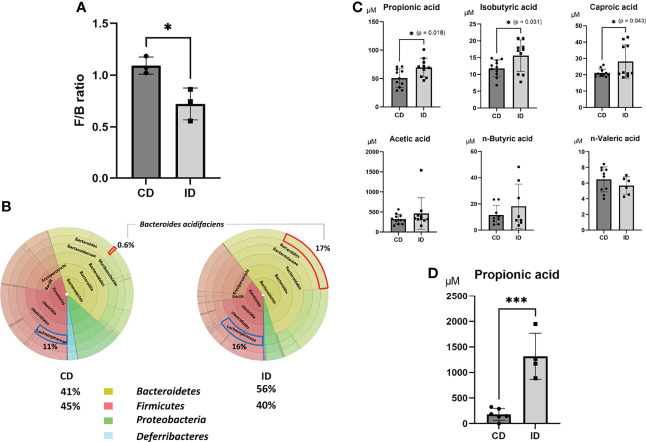
Effects of an inulin diet on the distribution of gut microbiota and the concentrations of short-chain fatty acids in the portal vein. The feces obtained from mice fed a CD or an ID were collected and analyzed by sequencing of the conserved 16S ribosomal RNA (rRNA) gene to examine gut microbiota. **(A)** The decreased ratio of *Firmicutes* (F) to *Bacteroidetes* (B) (F/B ratio) in the CD and ID groups (n = 3 mice/group) (Power calculation: 0.912). **(B)** The average value of the distribution of gut microbiota in the CD and ID groups (n = 3 mice/group). **(C)** The concentrations of various fatty acids in the portal vein in mice of both groups were measured (n = 10-11 mice/group; *P < .05) (Power calculation: Propionic acid; 1.000, Isobutyric acid; 0.998, Caproic acid; 0.996). **(D)** The concentration of propionic acid in the cecal contents in mice of both groups was measured (n = 4 mice/group; ***P < .01) (Power calculation: 1.000).

It has been reported that inulin is fermented by gut microbiota into degradation products such as SCFAs. To investigate first whether the ID affects the changes in gut microbiota, fecal samples collected from both groups of CD and ID were examined by sequencing the conserved 16S rRNA gene. Among the various changes in gut microbiota, elevated levels of *Bacteroidetes* (from 41% to 56% of the gut microbiota) and reduced levels of *Firmicutes* (from 45% to 40% of the gut microbiota) were observed in the ID group compared with the CD group (**Figure 2A**). Furthermore, the ratio of *Bacteroides acidifaciens*, a potential inulin utilizer (36), was significantly increased in feces of the ID group (from 0.6% to 17% of the gut microbiota) (**Figure 2B**). It has been reported that feeding mice with an inulin-rich diet resulted in the increase of *Bacteroides acidifaciens* in their feces (37). The decreased ratio of *Firmicutes* to *Bacteroidetes* (F/B) might affect SCFA production in the intestinal tract of mice. The majority of SCFAs is produced mainly by gut microbiota through the saccharolytic fermentation of carbohydrates (38). A decrease in the F/B ratio has been reported to be positively correlated with the fecal concentration of SCFAs, such as acetic acid, PA, and butyric acid (39). These SCFAs are supposed to be absorbed from the intestinal mucosa into the portal vein. Therefore, the concentrations of various fatty acids in the portal vein were measured in both groups (**Figure 2C**). Among the SCFAs examined, levels of propionic acid (PA) (p = 0.018) as well as isobutyric acid (p = 0.031), and caproic acid (p = 0.043) were elevated significantly in the portal vein of mice in the ID group compared to that in the CD group. On the other hand, among *Firmicutes* genus, the increase of *Lachnospiraceae* in feces was also evident upon the inulin diet (**Figure 2B**). It has been reported that the enhancement of propionate is correlated with favorable increases of *Lachnospiraceae* (40), suggesting they might also contribute to the increment of propionate induced by inulin diet. In fact, the concentration of PA in the cecal content obtained from mice fed with ID for 14 days was significantly higher than that from mice fed with CD (**Figure 2D**).

PICRUSt (Phylogenetic Investigation of Communities by Reconstruction of Unobserved States) analysis (29) was used to predict the abundances of functional genes in fecal microbiota of CD and ID groups of mice. In the Kyoto Encyclopedia of Genes and Genomes (KEGG) pathways, the microbial gene functions related to PA biosynthesis were much higher in the fecal microbiome of the ID group (p < 0.05, **Figure S2**). From these analyses, although the effect of butyric acid on the suppression of liver IRI has been well examined previously (17, 19, 21), we focused in the present study especially on the action of PA, which showed the most significant elevation in the portal vein in the ID group, as another possible candidate for the amelioration of mouse liver IRI.

### Intraperitoneal PA Administration Strongly Improved Liver IRI by Suppressing Inflammatory Cytokines

**Figure 3:**
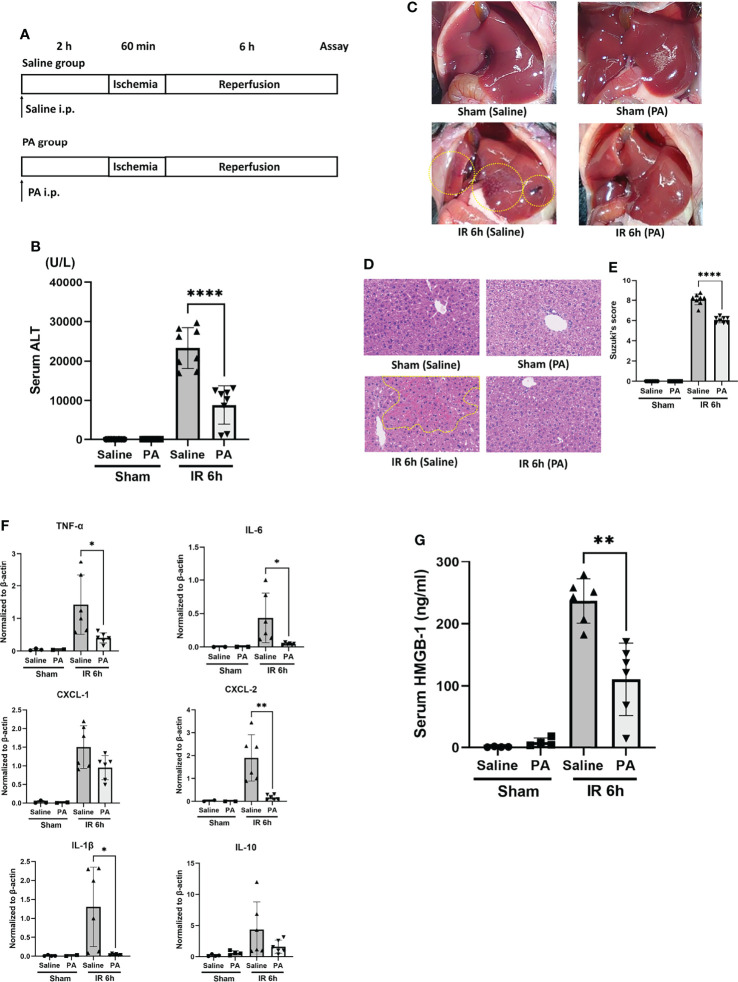
Intraperitoneal pretreatment with propionic acid ameliorates liver IRI in mice. **(A)** WT mice were divided into two groups. Mice in the PA group were intraperitoneally pretreated with PA (5 mmol/kg in saline) 2 h prior to liver ischemia, whereas mice in the saline group were pretreated with saline. Then, mice in both groups were performed liver IRI model. **(B)** The sALT level after 6 h of reperfusion (n = 8 mice/group; ****P < .0001) (Power calculation: 1.000). **(C)** Macroscopic findings after 6 h of reperfusion in saline- and PA -pretreated mice. Regions (bleeding and swelling) of macroscopic changes induced by IR insult are circled by dotted lines. **(D)** Representative liver histology (H&E staining) after IR insult (magnification ×400). Congestion area was shown in circle by yellow dot. **(E)** Suzuki’s histological grading (26) in each group (n = 8 mice/group; ****P < .0001) (Power calculation: 1.000). **(F)** Quantitative RT-PCR detection of inflammatory cytokines (TNF-α, IL-6, CXCL-1, CXCL-2, IL-1β, and IL-10) at 6 h of reperfusion. Data were normalized to β-actin gene expression (n = 6 mice/group; *P < .05, **P < .01) (Power calculation: TNF-α; 0.998, IL-6; 0.980, CXCL-2; 1.000, IL-1β; 0.996). The reduction of CXCL-1 and IL-10 in PA-treated group compared to saline-treated group was not significant. **(G)** The levels of serum HMGB-1 at 6 h after reperfusion in saline- and PA -pretreated mice (n = 8 mice/group; **P < .01) (Power calculation: 1.000).

In order to clarify whether or not the effect of PA plays a crucial role in the ID-induced amelioration of liver IRI, PA (at 5 mmol/kg, 100μM per a mouse of 20 grams weight, in saline) was administered intraperitoneally 2 hours before the ischemic insult as shown in **Figure 3A**. As a control, mice were intraperitoneally administered saline only instead of PA at the same time point. The sALT level was significantly improved in the PA group (**Figure 3B**). Macroscopically (**Figure 3C**) and microscopically (**Figure 3D**), PA-pretreated mice clearly showed less liver damage caused by IR insult, than saline-pretreated mice. Pathological findings (Suzuki’s score, **Figure 3E**) were also improved in the PA group. The expression of inflammatory cytokines and chemokines in liver tissues after IR stimulation was clearly suppressed in the PA group, that is, IL-6, TNF-α, IL-1β, and CXCL-2 but not CXCL-1 were remarkably reduced in the PA-treated group than in the saline-treated group (**Figure 3F**). However, similarly to the results of inulin-diet fed mice as shown in **Figure 1H**, the expression of IL-10, which is known as one of the immunosuppressive cytokines, was not affected by PA treatment, suggesting that immunoregulatory effect may not be involved in the suppression of inflammation induced by PA as well as the inulin. HMGB-1 has been reported as one of the DAMPs released from necrotic cells and hepatocytes in the case of liver IR insult, which leads to the activation of macrophages, followed by the production of proinflammatory cytokines (3, 8). Our previous study revealed that the HMGB-1/TLR-4 pathway played a critical role in the process of liver IRI (5, 6). To clarify how intraperitoneal PA administration affects the HMGB-1/TLR-4 pathway, HMGB-1 levels after the IR treatment were measured in the sera of PA and saline-injected mice. The serum HMGB-1 level was significantly increased 6 hours after reperfusion, whereas the level of HMGB-1 after IR was markedly reduced in the PA group (**Figure 3G**). The reduction of serum HMGB-1 by PA pretreatment might downregulate the further activation of immune cells through the TLR-4 pathway, resulting in the lowered expression of inflammatory cytokines and chemokines. These data indicate that PA reduces the release of HMGB-1 by suppressing liver cell injury or death caused by the IR insult, which results in the downregulation of the TLR-4 pathway, followed by the reduction of proinflammatory cytokines.

### PA Strongly Increased Acetylation of Histone-3 and Expression of Antioxidant Enzyme HO-1 in Liver Tissues After IR Stimulation

**Figure 4:**
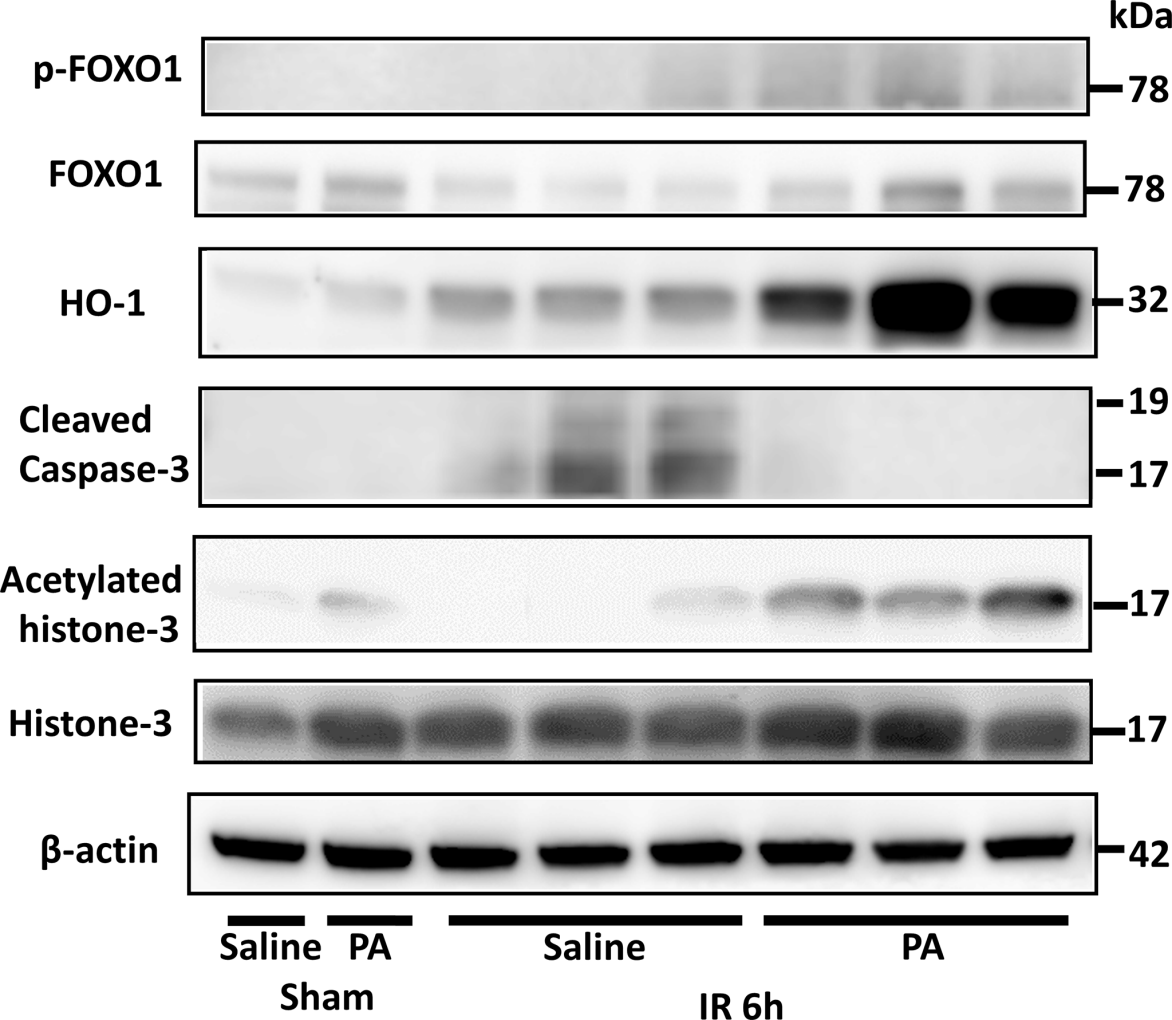
An antioxidative effect of PA on liver tissues after IR insult by inducing the acetylation of histone-3 and the expression of HO-1. Western blot assisted analyses of the total and phosphorylated protein levels of FOXO1, acetylated histone-3, histone-3, HO-1, and cleaved caspase-3 in liver tissues after 6 h of reperfusion. β-actin was used as the internal control.

Forkhead box protein O1 (FOXO1) is involved in the transcriptional regulation of antioxidant enzymes such as heme oxygenase 1 (HO-1) (41). It has been reported that HO-1 overexpressing cells increased survival against oxidative stress (42). As shown in **Figure 4**, at 6 hours after reperfusion, FOXO1 expression in the liver of PA (5 mmol/kg in saline)-pretreated mice was slightly higher than that of saline-pretreated mice. HO-1 expression increased more clearly in the liver of the PA-pretreated group. Besides, the increased expression of acetylated histone-3 was observed in the liver of the PA pretreated-group, indicating that the increase of histone-3 acetylation induced FOXO1 expression, followed by HO-1 upregulation, which increased liver cell survival against the ischemia-induced oxidative stress. As a result, the increased expression level of cleaved caspase-3 after IR was strongly downregulated and almost diminished by PA pretreatment. These results indicate that PA pretreatment exhibited antioxidative effects and suppressed the liver cell death caused by an IR insult.

### Preconditioning With PA Suppressed TNF-α Production *In Vitro* From Mouse Peritoneal Macrophages Stimulated by HMGB-1

**Figure 5:**
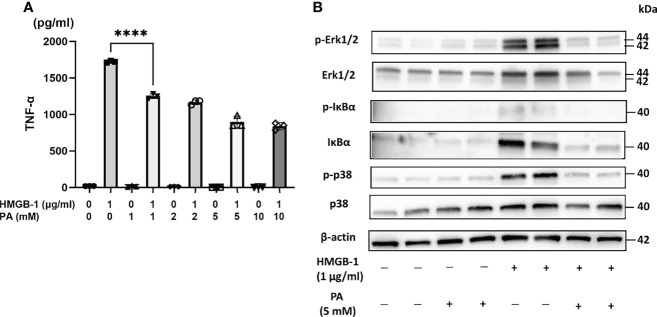
PA suppressed HMGB-1-mediated activation of peritoneal macrophages. Peritoneal macrophages from WT mice stimulated by HMGB-1 (1 µg/mL) were cultured with or without the pretreatment with PA (1, 2, 5, and 10 mM, respectively) for 48 h. **(A)** TNF-α release was measured by ELISA (n = 3 samples/group; ****P < .0001) (Power calculation: 1.000). **(B)** Western blot assisted analyses of NF-κB signaling and MAPK signaling molecules in the macrophages stimulated by HMGB-1 (1 µg/mL) 30 min after the pretreatment with PA (5 mM). β-actin was used as the internal control.

To clarify the direct effect of PA on HMGB-1-induced production of proinflammatory cytokines such as TNF-α from immune cells, the mouse peritoneal macrophages were stimulated *in vitro* with HMGB-1 in the presence or absence of PA. At first, peritoneal macrophages from wild-type mice were cultured in RPMI 1640 medium containing various concentrations of PA (0-10 mM). Thirty minutes after the pretreatment with PA, bovine HMGB-1 (1 µg/mL) was added. As shown in **Figure 5A**, in the case of macrophages without PA pretreatment, the stimulation with HMGB-1 resulted in the significant elevation of TNF-α secretion. By contrast, in the case of macrophages pretreated with PA, the TNF-α secretion was markedly downregulated in a PA dose-dependent manner from 1 mM. As shown in **Figure 5B**, the expressions of canonical nuclear factor kappa B (NF-κB) signaling and mitogen-activated protein kinase (MAPK) signaling molecules, such as IκBα, Erk1/2, p-Erk1/2, and p-p38, were markedly decreased in the PA-pretreated macrophages. These results indicate that PA not only causes the reduction of HMGB-1 released from IR-affected cells but could also directly inhibit the TLR-4 mediated inflammatory responses in macrophages.

## Discussion

It is now well known that gut flora affects the host’s overall health. The human gut microbiota includes two major phyla, *Bacteroidetes* (B) and *Firmicutes* (F). As reported previously, the fecal concentration of propionate, one of SCFAs, is correlated positively with *Bacteroidetes* (43) and the F/B ratio was negatively correlated with fecal levels of propionate and butyrate in healthy humans (44). Another report showed that in case of mice, aging increases the relative abundance of *Firmicutes* while decreasing that of *Bacteroidetes*. Young mice transplanted with the supernatant of fecal suspensions from aged mice with high F/B ratio had a greater increase in proinflammatory cytokines following a stroke, compared to the aged mice transplanted with microbiota from young mouse fecal suspension of low F/B ratio (45). These reports suggest that manipulating the gut microbiota results in the changes of SCFA production and affects the activities of immune cells. SCFAs absorbed from the intestinal mucosa are supposed to flow into the portal vein stream further into liver. It has been reported that gut microbiota-derived propionate reduced cancer cell proliferation in the liver (13, 46). It was reported that feeding mice with an inulin-rich diet resulted in the increase of *Bacteroides acidifaciens* in their feces and in the increased radiosensitivity of the mouse tumors (37).

The present study showed that diet of inulin, a water-soluble nondigestible carbohydrate dietary fiber, elevated levels of *Bacteroidetes* and reduced levels of *Firmicutes*. Inulin diet resulted also in the increase of *Bacteroides acidifaciens* in feces (**Figure 2**). This result is consistent with a previous report which demonstrated that inulin-rich diet elevated levels of acetic, propionic and butyric acids in mouse feces and increased plasma PA levels in mice (47). The present study provides clear evidence that inulin diet significantly increased propionate, one of the SCFAs, and showed a protective effect against warm liver IRI in mice. Further, it was clearly shown that the direct intraperitoneal administration of PA alone also efficiently ameliorated liver IRI in mice by preventing apoptotic cell death of hepatocytes as well inflammatory responses. PA also strongly suppressed *in vitro* activation of macrophages induced by HMGB-1 through TLR-4. As demonstrated in **Figure 4**, intraperitoneal PA administration remarkably increased the upregulation of acetylated histone-3, FOXO1, and HO-1. It has been reported that sodium propionate inhibited histone deacetylases (HDACs) 2 and 8 activity in bovine mammary epithelial cells, which led to the increase of histone-3 acetylation (48). The intraperitoneal administration of PA in mice might result in the inhibition of HDACs, followed by the increased acetylation of histone-3 in liver tissues, which protected mice from liver injury caused by IR. It has been reported that acetylated histone induced by the HDAC inhibitor increases the FOXO1 protein level and its target gene (49). FOXO1 regulates the expression of HO-1 by binding to the promoter of HO-1 (41). HO-1 is a member of the heat shock protein family, which is associated with cellular antioxidant defense and antiapoptotic functions. HO-1 knockdown is reported to enhance heat stress-induced ROS generation and apoptosis, which reduces the antioxidative responses (50). Furthermore, it has been reported that postoperative but not preoperative liver HO-1 expression correlates negatively with IRI severity in patients with orthotopic liver transplantation (51). Thus, the upregulation of HO-1 expression in liver tissues pretreated with PA as shown in **Figure 4** may play an essential role in the amelioration of liver IRI. Antioxidative nutrient-rich diets, including vitamins C and E, could partially suppress liver injury by reducing ROS production (52). An inulin-rich diet could similarly and more efficiently ameliorate IR-induced liver damage by antioxidative effects by modulating gut microbiota and increasing PA in the portal vein.

HMGB-1 is a molecule released from necrotic cells. It has been reported that the blockade of HMGB-1 by anti-HMGB-1 antibody could protect liver IRI (53, 54). We have reported that recombinant thrombomodulin and its derivative lectin-like domain could inhibit the HMGB-1 and TLR-4 interaction, which resulted in the suppression of mouse liver IRI (5, 6). As shown in **Figure 3G**, serum HMGB-1 upregulated by liver IR was decreased significantly by the PA pretreatment probably because of the suppression of liver cell death, as stated above. This reduction of serum HMGB-1 might result in the reduction of inflammatory responses transmitted through the HMGB-1 and TLR-4 signal pathway.

The question arises as to whether PA exhibits a suppressive effect on the HMGB-1/TLR-4 pathway in mouse liver IRI not only by the reduction of TLR-4 ligand, HMGB-1, but also through a direct action on the TLR-4 signal cascade. High-dose propionate has been reported to reduce LPS-induced inflammatory response in mouse endothelial cells and alveolar macrophages in a TLR-4 dependent manner (55). Another report showed the protective effect of PA against IκBα degradation and p65 translocation in LPS-treated IEC-6 cells (56), which suggests the suppression of the NF-κB/TLR-4 pathway by PA. In the present study, PA directly suppressed the HMGB-1 triggered TLR-4 activation in macrophages and suppressed TNF-α production. The amelioration of liver IRI by the pretreatment with inulin or PA might be achieved not only by blocking the death of liver cells but also by the suppression of the inflammation in macrophages through downregulation of TLR-4/NF-κB as well as MAPK pathways. It has been reported that propionate showed an immune-modulatory role against hypertensive cardiac damages and significantly attenuated cardiac hypertrophy, fibrosis, vascular dysfunction, and hypertension (57).

In conclusion, inulin pretreatment can alleviate liver IRI in mice. This protective effect is achieved firstly by the antioxidative and antiapoptotic effects on liver cells through the increase of PA in the portal vein, which activated the acetylated histone-3/FOXO-1/HO-1 pathway. This resulted in the inhibition of cell death and the reduction of HMGB-1 released from necrotic cells. Besides, the suppression of inflammatory cytokines through the direct action of PA on inflammatory cells such as macrophages also contributed to the amelioration of mouse liver IRI.

## Data Availability Statement

The original contributions presented in the study are publicly available. This data can be found here: NCBI, BioProject, PRJDB13048.

## Ethics Statement

The animal study was reviewed and approved by the Animal Research Committee of The Tazuke Kofukai Medical Research Institute, Kitano Hospital, Osaka, Japan.

## Author Contributions

JK, TM, and HK performed experiments. JK, YU, TM, and TW planned the study and analyzed data. JK, YU, HK, and TW wrote the manuscript. All authors contributed to, read and approved the final manuscript.

## Funding

This study was supported by a Grant-in-Aid for Scientific Research C: 18K08609, B: 20H03743 and Research Activity Start-up: 19K24019 from the Ministry of Education, Culture, Science and Sports, Japan and by the Kitano Research Grant from The Tazuke Kofukai Medical Research Institute, Kitano Hospital.

## Conflict of Interest

The authors declare that the research was conducted in the absence of any commercial or financial relationships that could be construed as a potential conflict of interest.

## Publisher’s Note

All claims expressed in this article are solely those of the authors and do not necessarily represent those of their affiliated organizations, or those of the publisher, the editors and the reviewers. Any product that may be evaluated in this article, or claim that may be made by its manufacturer, is not guaranteed or endorsed by the publisher.

## Supplementary Material

Supplementary Figure 1 The ratio of Foxp3-positive cells to CD4-positive cells in mesenteric lymph nodes in CD and ID group mice. (n = 5 mice/group).

Supplementary Figure 2 PICRUSt2 analysis in the MetaCyc pathways for the fecal microbiome (24). Significant MetaCyc pathways for the fecal microbiome of the ID group and the CD group were identified by STAMP software. Rounded square highlights data showing the enhanced propionate biosynthesis from pyruvate in the gut microbiome of the ID group. PICRUSt, Phylogenetic Investigation of Communities by Reconstruction of Unobserved States; STAMP, Statistical Analysis of Metagenomic Profiles.

Supplementary Table 1 List of antibodies used for western blotting analysis.

Supplementary Table 2 List of primer couples generated for qRT-PCR.

## Abbreviations

CD, control ordinary diet; DAMPs, damage-associated molecular patterns; ELISA, enzyme-linked immunosorbent assay; ERAS, Enhanced Recovery After Surgery; F/B, Bacteroidetes-to-Firmicutes; FOXO1, Forkhead box protein O1; GPR, G protein-coupled receptor; HDAC, histone deacetylase; HMGB-1, high-mobility group box 1 protein; HO-1, heme oxygenase-1; ID, inulin diet; IR, ischemia and reperfusion; IRI, ischemia and reperfusion injury; PA, propionic acid; ROS, reactive oxygen species; sALT, serum alanine aminotransferase; SCFA, short-chain fatty acid; STAT, signal transducer and activator of transcription; TLR-4, Toll-like receptor 4; TNF-α, tumor necrosis factor-α; Treg cells, regulatory T cells; TUNEL, terminal deoxynucleotidyl transferase-mediated deoxyuridine triphosphate nick-end labeling; WT, wild-type.
