# Supplementary figures and images for: Propionic Acid, Induced in Gut by an Inulin Diet, Suppresses Inflammation and Ameliorates Liver Ischemia and Reperfusion Injury in Mice
Source: Front Immunol. 2022 Apr 22;13:862503. doi: 10.3389/fimmu.2022.862503 (PMC9097600; doi:10.3389/fimmu.2022.862503)

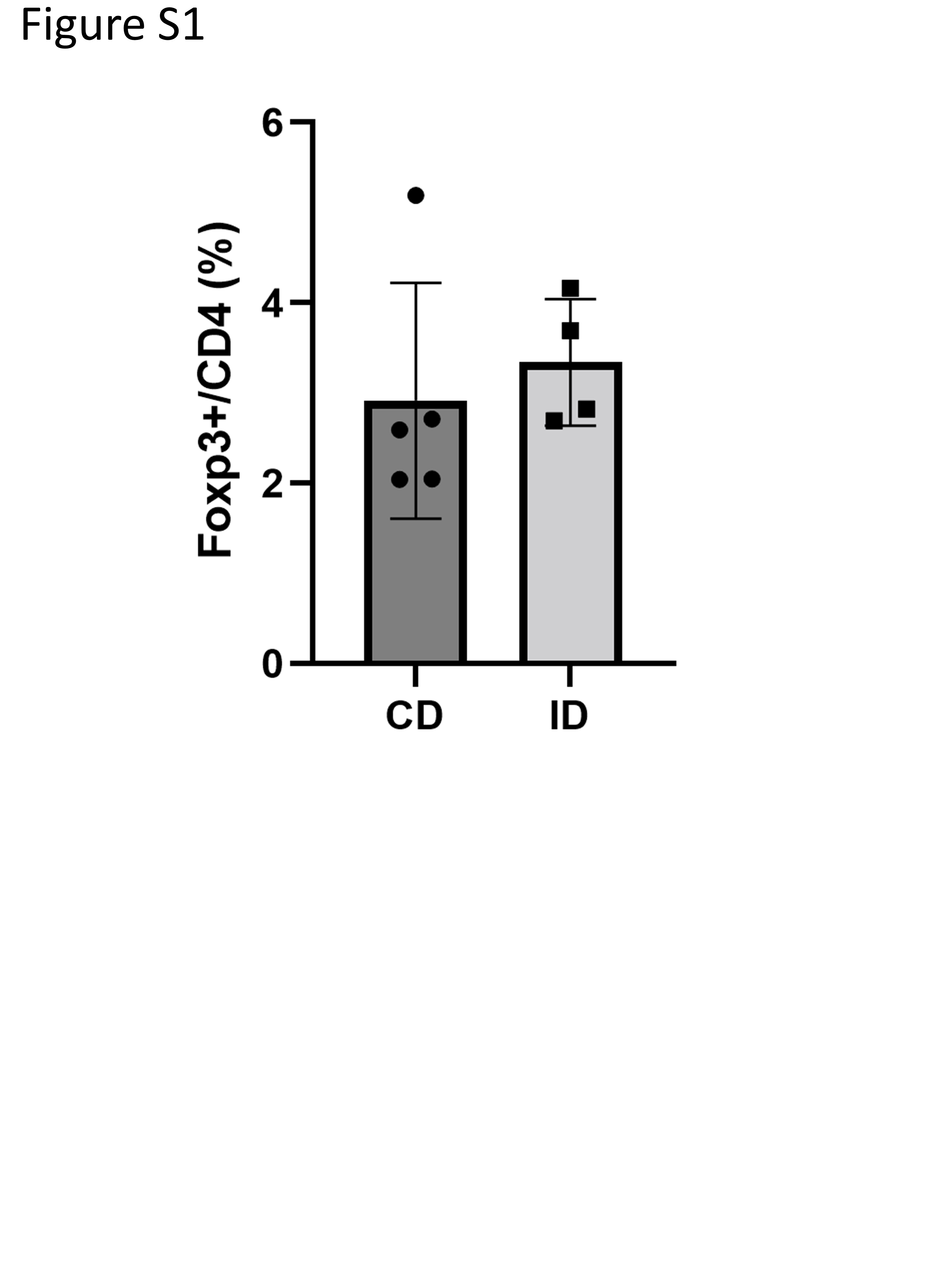

Supplement: Supplementary Figure 1 — The ratio of Foxp3-positive cells to CD4-positive cells in mesenteric lymph nodes in CD and ID group mice. (n = 5 mice/group). [file Image_1.tif]

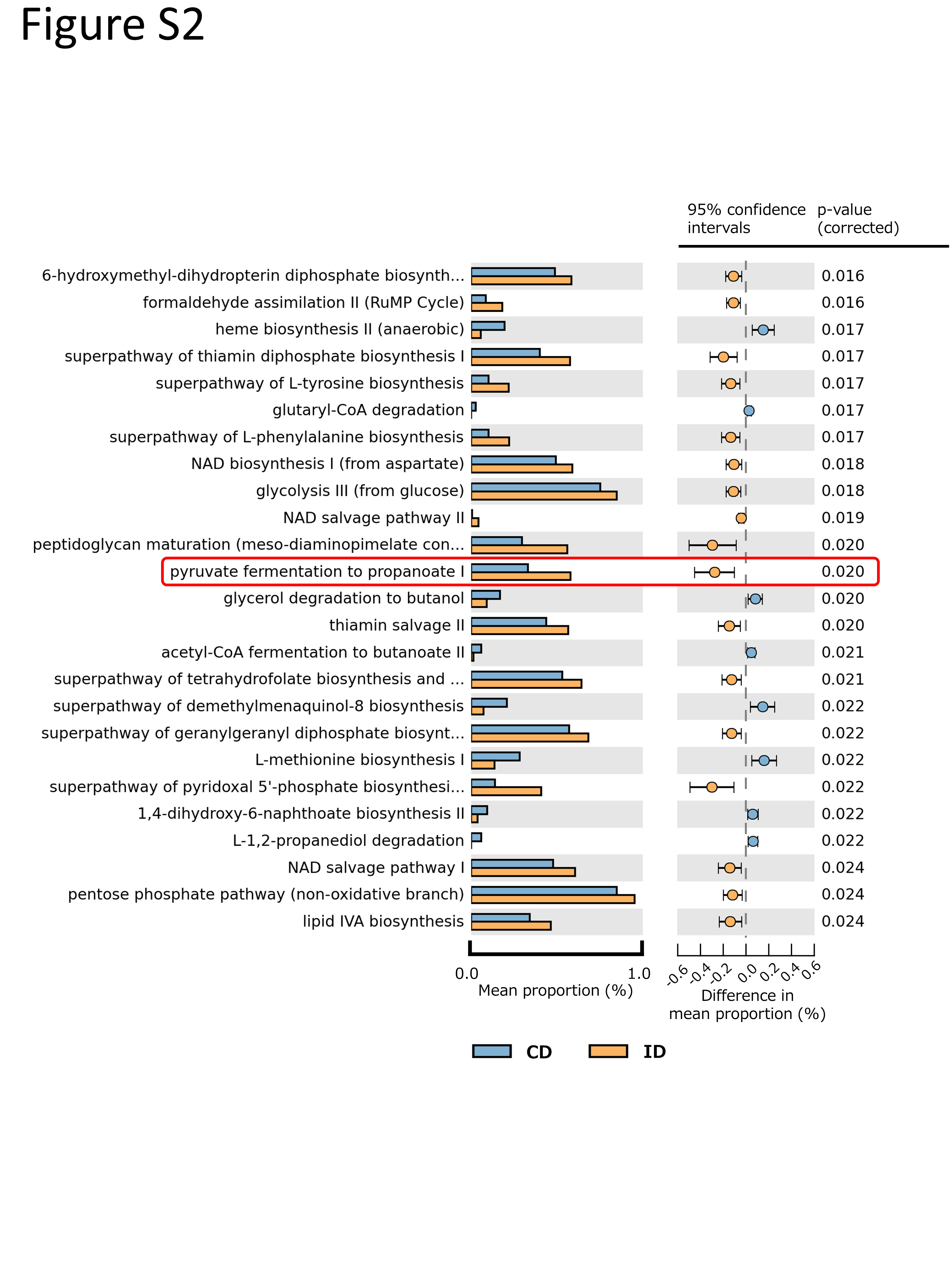

Supplement: Supplementary Figure 2 — PICRUSt2 analysis in the MetaCyc pathways for the fecal microbiome (24). Significant MetaCyc pathways for the fecal microbiome of the ID group and the CD group were identified by STAMP software. Rounded square highlights data showing the enhanced propionate biosynthesis from pyruvate in the gut microbiome of the ID group. PICRUSt, Phylogenetic Investigation of Communities by Reconstruction of Unobserved States; STAMP, Statistical Analysis of Metagenomic Profiles. [file Image_2.tif]

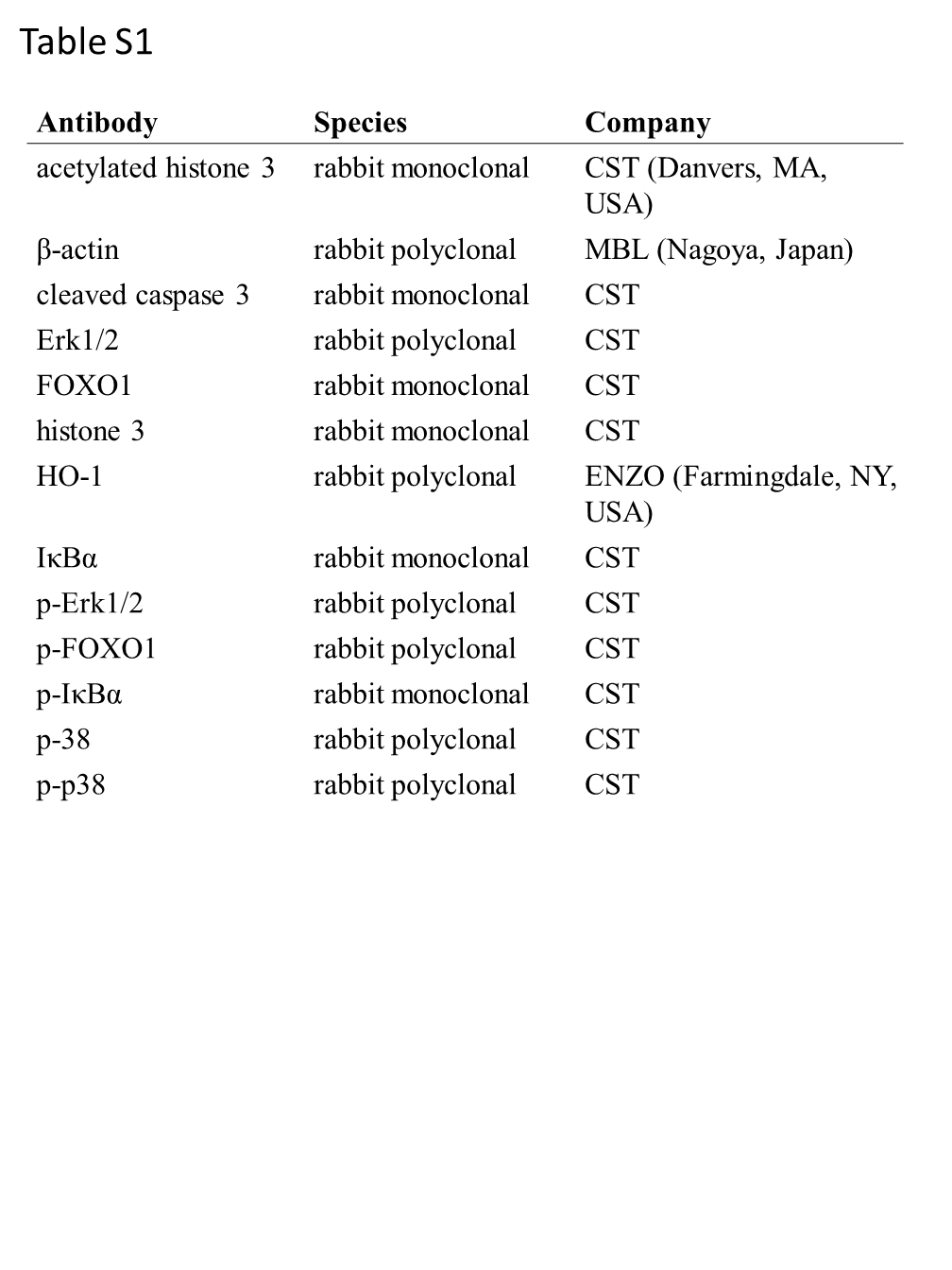

Supplement: Supplementary Table 1 — List of antibodies used for western blotting analysis. [file Image_3.tif]

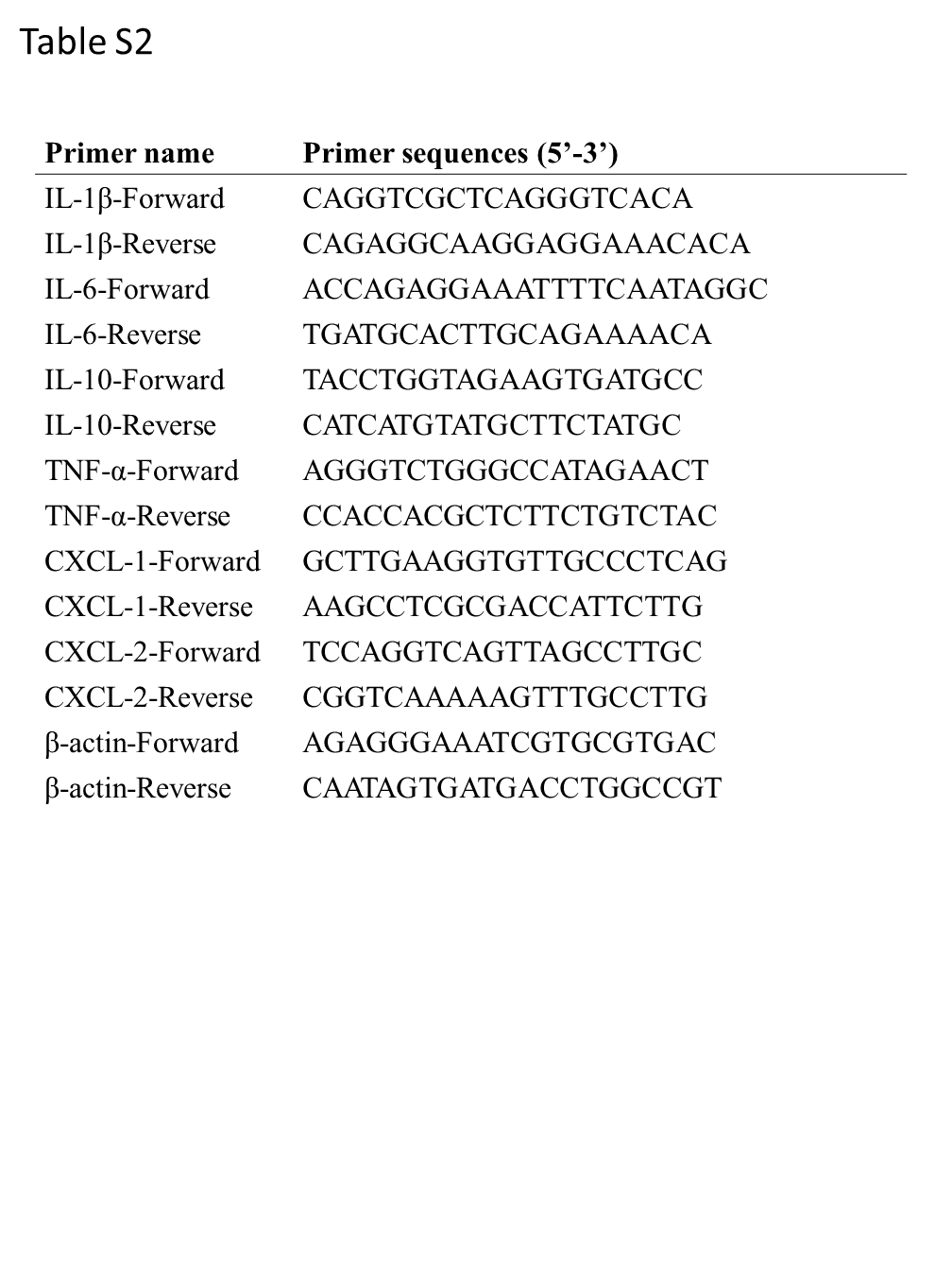

Supplement: Supplementary Table 2 — List of primer couples generated for qRT-PCR. [file Image_4.tif]
